# Role of Brown and Beige Adipose Tissues in Seasonal Adaptation in the Raccoon Dog (*Nyctereutes procyonoides*)

**DOI:** 10.3390/ijms22179623

**Published:** 2021-09-06

**Authors:** Laura Niiranen, Kari A. Mäkelä, Shivaprakash J. Mutt, Riikka Viitanen, Anna Kaisanlahti, David Vicente, Tommi Noponen, Anu Autio, Anne Roivainen, Pirjo Nuutila, Seppo Saarela, Karl-Heinz Herzig

**Affiliations:** 1 Research Unit of Biomedicine, Faculty of Medicine, University of Oulu, P.O. Box 5000, FI-90014 Oulu, Finland; laura.niiranen@oulu.fi (L.N.); kari.makela@oulu.fi (K.A.M.); shivaprakash.jagalurmutt@mcb.uu.se (S.J.M.); 2 Turku PET Centre, University of Turku, FI-20520 Turku, Finland; ralsii@utu.fi (R.V.); anu.autio@utu.fi (A.A.); aroivan@utu.fi (A.R.); pirjo.nuutila@utu.fi (P.N.); 3 Cancer and Translational Medicine Research Unit, Faculty of Medicine, University of Oulu, FI-90014 Oulu, Finland; anna.kaisanlahti@oulu.fi; 4 Biocenter Oulu, University of Oulu, FI-90014 Oulu, Finland; 5 Veterinary Hospital Mevet, 00380 Helsinki, Finland; david.g.vicente@gmail.com; 6 Department of Clinical Physiology and Nuclear Medicine, Turku University Hospital, FI-20520 Turku, Finland; Tommi.Noponen@tyks.fi; 7 Department of Medical Physics, Turku University Hospital, FI-20520 Turku, Finland; 8 Turku PET Centre, Turku University Hospital, FI-20520 Turku, Finland; 9 Turku Centre for Disease Modeling, Finland Department of Medical, University of Turku, FI-20520 Turku, Finland; 10 Ecology and Genetics Research Unit, Faculty of Science, University of Oulu, FI-90014 Oulu, Finland; seppoyo.saarela@gmail.com; 11 Medical Research Center Oulu, FI-90014 Oulu, Finland; 12 Oulu University Hospital, FI-90014 Oulu, Finland; 13 Institute of Pediatrics, Poznań University of Medical Sciences, 60-572 Poznań, Poland

**Keywords:** brown adipose tissue, browning, beige/brite adipocytes, UCP1, seasonal obesity, winter sleep/hibernation, thermoregulation, seasonal adaptation

## Abstract

Brown adipose tissue (BAT) expresses uncoupling protein-1 (UCP1), which enables energy to be exerted towards needed thermogenesis. Beige adipocytes are precursor cells interspersed among white adipose tissue (WAT) that possess similar UCP1 activity and capacity for thermogenesis. The raccoon dog (*Nyctereutes procyonoides*) is a canid species that utilizes seasonal obesity to survive periods of food shortage in climate zones with cold winters. The potential to recruit a part of the abundant WAT storages as beige adipocytes for UCP1-dependent thermogenesis was investigated in vitro by treating raccoon dog adipocytes with different browning inducing factors. In vivo positron emission tomography/computed tomography (PET/CT) imaging with the glucose analog ^18^F-FDG showed that BAT was not detected in the adult raccoon dog during the winter season. In addition, *UCP1* expression was not changed in response to chronic treatments with browning inducing factors in adipocyte cultures. Our results demonstrated that most likely the raccoon dog endures cold weather without the induction of BAT or recruitment of beige adipocytes for heat production. Its thick fur coat, insulating fat, and muscle shivering seem to provide the adequate heat needed for surviving the winter.

## 1. Introduction

Animal species inhabiting areas with dramatic changes in food availability and harsh environmental conditions can resort to seasonal obesity as a survival strategy [1]. In addition, adipose tissue may contribute to maintaining body heat during the cold winter seasons via beige/brite adipocytes. Adipose tissue is categorized into WAT, which functions mainly as an energy storage and secretory organ, and into the metabolically highly active BAT with thermogenic capacity [2,3]. BAT expresses UCP1, which uncouples the mitochondrial oxidative phosphorylation from ATP synthesis, resulting in the dissipation of energy as heat [4]. In addition, beige [5] or brown-in-white i.e., brite [6], adipocytes are scattered within WAT in rodents and in humans. Both BAT and beige adipocytes share a similar morphology with multilocular lipid droplets as compared to the unilocular lipid droplet in WAT [6,7]. The beige adipocytes are suggested to arise from the same non-myogenic cell precursor cells as WAT, whereas BAT is derived from the myogenic Myf5 (myogenic factor 5) encoding precursor cells [6]. Similar to BAT, beige adipocytes have also a high mitochondrial content and express BAT genes, most notably the thermogenic *UCP1*. However, beige adipose tissue also expresses specific genes (e.g., Aqp7, aquaporin 7; Asc1, Guanine nucleotide-binding protein subunit beta-like protein; Car4, Carbonic anhydrase 4; Tbx1, T-Box Transcription Factor 1) [8], making it a distinct adipose tissue type, despite the shared characteristics with both WAT and BAT [6,9]. The signals triggering BAT activation and the recruitment of beige adipocytes in the “browning process” are under great research interest due to the potential implications for metabolic research. The main route of signaling leading to UCP1 activation in BAT and beige adipose tissue is cold-induced noradrenaline release (NA) from the sympathetic nerve terminals and their binding to β-adrenergic receptors (β-ARs), leading to an increase in free fatty acids and uncoupling of the respiratory chain in the mitochondria [10,11]. In addition to β-AR agonists (e.g., NA and synthetic agonists), peroxisome proliferator activated receptor (PPAR) ligands (e.g., fenofibrate), hormones (e.g., insulin and thyroid hormones), thiazolidinediones (e.g., rosiglitazone), natriuretic peptides (e.g., ANP, atrial natriuretic peptide; BNP, B-type natriuretic peptide), metabolites (e.g., lactate, β-hydroxybutyrate), inflammation, and exercise related cytokines (e.g., irisin) have been found to induce beige adipocytes [12,13,14].

The recruitment of WAT into beige adipocytes has been studied extensively in human and mouse adipocytes, but the underlying signaling mechanisms and the impact of the increased energy expenditure in the form of heat production by beige adipocytes during seasonal changes remains unclear. Animals that are subjected to non-pathological body weight cycles according to seasonal phases and the environmental temperature changes offer an interesting model to study the signals triggering WAT browning and the physiological relevance of beige adipose tissue. Our model species, the raccoon dog, is a middle-sized omnivorous canid species that undergoes repeated and drastic seasonal body weight and adiposity fluctuations [15]. Raccoon dogs can almost double their weight with the accumulated body fat storages after summer as they prepare for the winter food scarcity. During the challenging winter conditions, the raccoon dogs rely mostly on their body fat storages for energy and can endure long fasting periods [16]. The dramatically opposite conditions of autumnal fattening and winter fasting are both well-tolerated [17,18]. The raccoon dog can also enter a light hypometabolic state to preserve energy. During the winter sleep, the raccoon dog undergoes a relatively shallow hypothermia (body temperature (T_B_) reduction only 0.5–1.5 °C) as compared to other winter sleeping species, e.g., the brown bear (*Ursus arctos arctos*), which reduces its’ T_B_ by 4–5 °C [15,19]. Hibernating animals undergo a more complete metabolic depression and T_B_ reduction [20], and BAT activity decreases as the body temperature is reduced in order to change to the energy-saving hibernation T_B_ in the colder climate. BAT is activated during hibernation, if the temperature of the environment falls below 0 °C, in order to maintain the T_B_ set-point as heat production by muscle activity or shivering cannot fully be utilized during hibernation [21]. In hibernating animals, BAT is proposed to be more relevant in the arousal from hibernation [22,23].

In the present study, we investigated the functional role of BAT and the potential recruitment of beige adipocytes for UCP1-dependent thermogenesis in an animal model undergoing seasonal temperature changes and non-pathological obesity. Our hypothesis proposes that a part of the abundant WAT storages in the raccoon dog will be recruited into beige adipocytes with induction of UCP1. The main objectives of the study were to decipher the role of BAT in thermoregulation, the browning capacity of raccoon dog WAT and the potential signals triggering this transformation.

## 2. Results

### 2.1. Whole-Body Distribution of Glucose Analogue ^18^F-FDG Uptake with PET/CT Imaging

**Figure 1:**
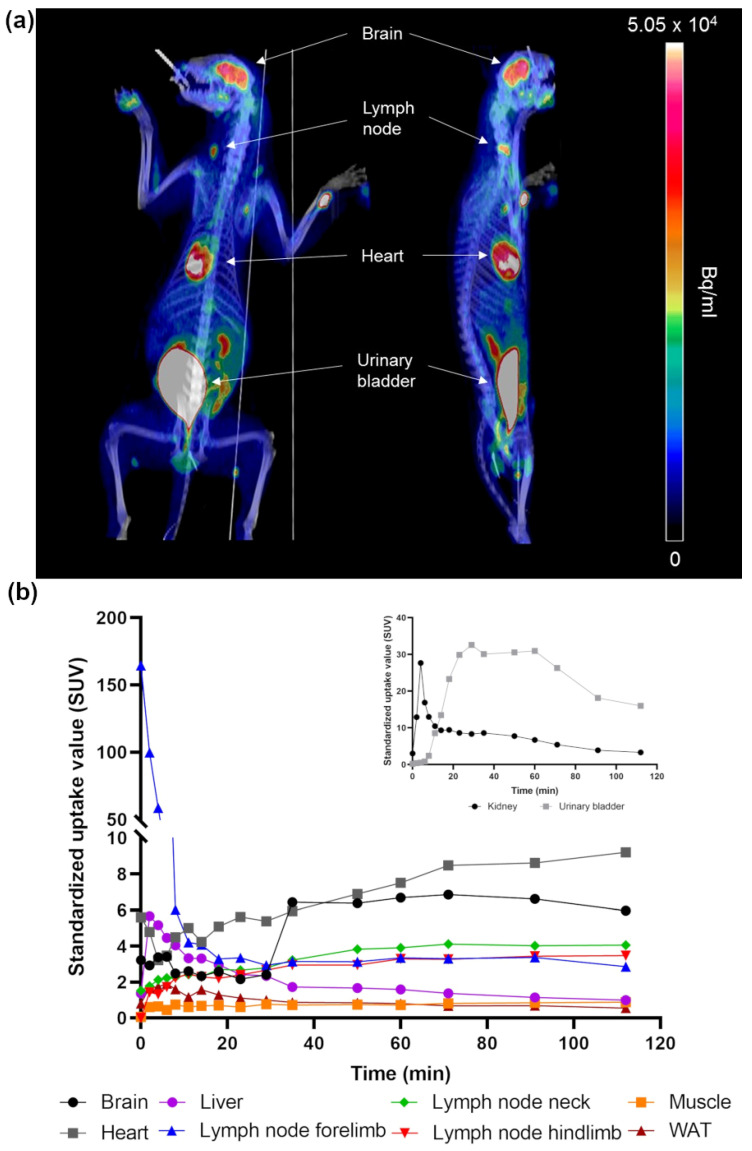
(**a**) Representative coronal and sagittal PET/CT images of one of the raccoon dogs. PET image represents an average radioactivity concentration during 120 min imaging. The highest radioactivity concentration is observed in brain, heart, urinary bladder, and lymph nodes in neck and forelimb. (**b**) Corresponding radioactivity concentration as a function of time from selected tissues.

Three raccoon dogs were successfully PET/CT imaged for whole-body distribution of glucose analog fluorodeoxyglucose F 18 (^18^F-FDG) uptake up to 2 h. ^18^F-FDG acts as a marker for glucose consumption and the analog accumulates at the sites of high metabolism, reflecting the activity of the tissue or organ. The ^18^F-FDG uptake was clearly detected in brain, lymph nodes, heart, kidneys, and urinary bladder (Figure 1a). Quantitative analysis of PET images illustrated the uptake kinetics of ^18^F-FDG in different tissues as time-activity curves (Figure 1b). BAT was not visualized in the ^18^F-FDG PET/CT. In vivo PET/CT images revealed that several lymph nodes were metabolically active. Samples with ^18^F-FDG uptake in the PET images were evaluated histologically by hematoxylin-eosin staining of paraffin sections to identify BAT. The metabolic active tissues were identified as WAT, skeletal muscle, and lymph nodes (Supplementary Figure S1: Histological hematoxylin-eosin staining). Tissue radioactivity measurements confirmed the highest activity in the scapular region tissue to arise from lymph nodes.

**Figure 2:**
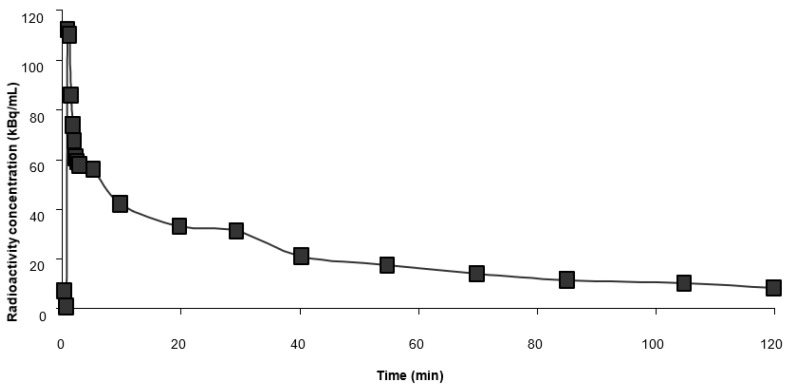
Representative plasma time-activity curve of the study from one of the raccoon dogs.

The analysis of PET images was in line with quantitative evaluations. When fractional uptake of ^18^F-FDG was analyzed using plasma time-activity (Figure 2) and tissue tracer uptake over scanning time, the highest uptake rates were in the brain and heart. The fractional uptake was elevated in lymph nodes (4–5–fold increase compared with skeletal muscles) and lowest in WAT (Table 1). Plasma glucose concentration was between 5–6 mmol/L in the animals during the study.

### 2.2. Dose- and Time-Dependents Treatment with Nonselective β-AR on UCP1 mRNA Expression of Primary Raccoon Dog Adipocytes

**Figure 3:**
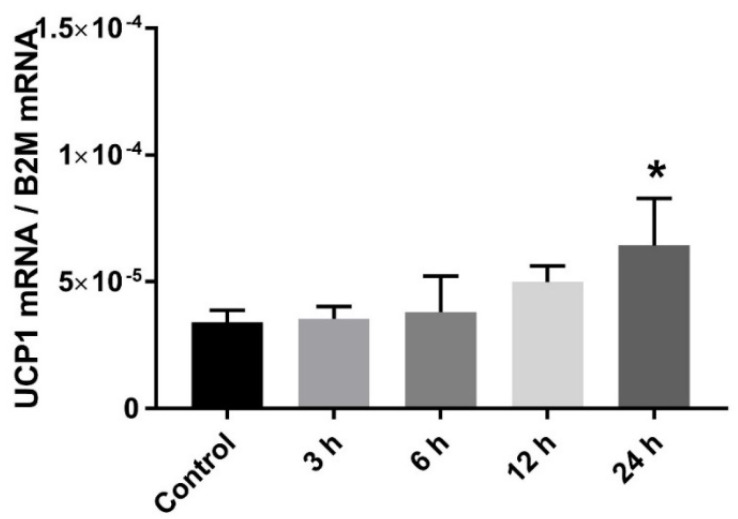
Relative expression of *UCP1* in raccoon dog adipocytes (*n* = 4) with 10 µmol/L isoprenaline for different time periods (3 h, 6 h, 12 h, and 24 h). Control adipocytes were treated with PBS. *UCP1* expressions are normalized to the expression of beta-2 microglobulin (*B2M)* and the result values are presented as mean ± SD. Statistically significant difference is indicated by * *p* < 0.05.

In order to further investigate our in vivo results, we established an in vitro system of the raccoon dog adipocytes and evaluated the dose-and time-dependent effects of the nonselective β-AR agonist isoprenaline on the relative *UCP1* expression. Raccoon dog adipocytes treated with isoprenaline in a dose-dependent manner with five concentrations (0.1 µmol/L, 1 µmol/L, 10 µmol/L, 100 µmol/L, and 1000 µmol/L) for 6 h [6,24] did not significantly increase *UCP1* expression (data not shown). 10 µmol/L isoprenaline induced the strongest response, while higher isoprenaline concentrations (100 µmol/L and 1000 µmol/L) induced lipolysis. 10 µmol/L isoprenaline was then utilized for the time-dependent treatment of the adipocytes (3, 6, 12, and 24 h). The relative *UCP1* expression was increased after 24-h treatment by 88.6% compared with control (*p* = 0.013, Figure 3), but no differences were found at 3, 6, and 12-h treatment (*p* > 0.05).

### 2.3. Chronic Treatment with Various Beige Adipose Tissue Inducers of UCP1 mRNA Expression in Raccoon Dog Primary Adipocytes

**Figure 4:**
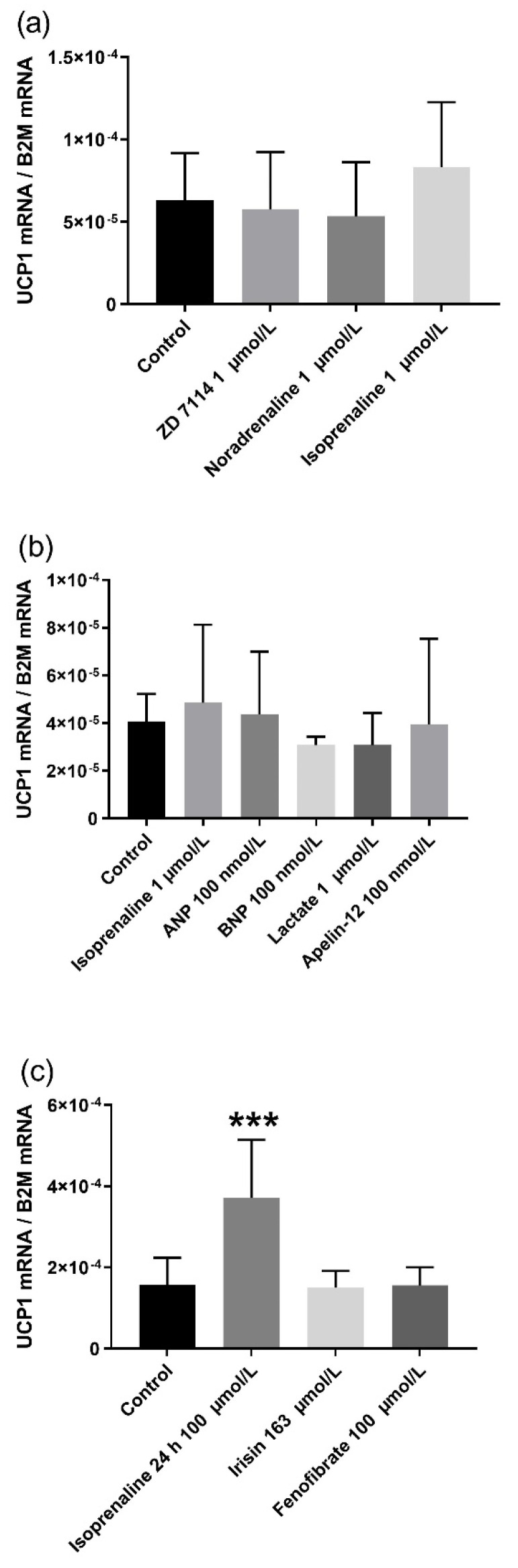
Relative expression of *UCP1* in raccoon dog adipocytes treated for 7 days with (**a**) 1 µmol/L ZD 7114 hydrochloride (ZD 7114), 1 µmol/L noradrenaline, and 1 µmol/L isoprenaline (*n* = 4). (**b**) 100 nmol/L ANP, 100 nmol/L BNP, 1 µmol/L lactate, and 100 nmol/L apelin-12 (*n* = 4). (**c**) Acute 24 h 100 µmol/L isoprenaline as positive control, 163 µmol/L irisin, and 100 µmol/L fenofibrate (*n* = 8). Control adipocytes were treated with PBS. *UCP1* expressions are normalized to the expression of *B2M* and the result values are presented as mean ± SD. Statistically significant difference is indicated by *** *p* ≤ 0.001.

The potential recruitment of white adipocytes to thermogenic beige adipocytes was further tested by chronic treatment of differentiated raccoon dog adipocytes with sympathetic activity-enhancing β-AR agonists and known browning-inducing factors. Chronic treatments did not change the relative *UCP1* expression in the raccoon dog adipocytes (Figure 4). Acute treatment with higher concentration of isoprenaline (100 µmol/L for 24 h) served as positive control and increased relative *UCP1* expression by 136.7% (*p* = 0.000) compared with control (Figure 4c).

### 2.4. Chronic Treatment with Various Beige Adipose Tissue Inducers of UCP1 mRNA Expression in Differentiated Mouse 3T3-L1 Adipocytes

To validate that our browning factor is indeed inducing *UCP1* expression, we used the mouse 3T3-L1 preadipocyte cell line as positive control. 3T3-L1 cells were treated with nonselective β-AR agonist isoprenaline, apelin, lactate, bone morphogenic protein 7 (BMP7), and irisin/fibronectin type III domain-containing protein 5 (FNDC5). Relative *UCP1* expression was significantly increased with 5 µmol/L isoprenaline by 303.5% (*p* = 0.000) and with 50 mmol/L lactate by 168.5% (*p* = 0.008) compared with control (Supplementary Figure S2. Relative expression of UCP1 in mouse 3T3-L1 cells).

## 3. Discussion

BAT has an important role in the thermogenesis of mammals after birth and in adult life in cold environments. Beige adipose tissue includes cells interspersed among WAT that possess capacity for UCP1 activity and can be recruited from the precursor cells scattered in WAT to produce heat in response to physiological signals. The raccoon dog gathers abundant WAT storages in summer and autumn to serve as an energy storage during the cold winter season, which could represent a potential reservoir for beige adipose tissue recruitment in the winter season [25]. Obesity and the related comorbidities are increasing at an alarming rate with adverse consequences to common human health and subsequently economy [26,27]. The possibility of the abundant WAT turning into energy-dissipating beige adipocytes by browning in the raccoon dog could function as a model and offer a broader perspective in the research of metabolic control in general.

Our in vivo results from the ^18^F-FDG PET/CT scan during the winter season indicate that the imaged adult raccoon dogs did not have BAT. The areas of active glucose metabolism in the scan were brain, myocardium, kidney, and lymph nodes. In contrast, glucose uptake in WAT was low, indicating that it did not contain BAT. Although ^18^F-FDG PET/CT has known limitations, especially when data is analyzed using standardized uptake values (SUVs, please see method section), it is the most commonly used technique for studying BAT. The more advanced Patlak graphical analysis, used in this study, combined with the correction for plasma glucose concentration, provides a more accurate quantification of tissue glucose utilization [28]. In addition to ^18^F-FDG, BAT accumulates a range of radiotracers, including other metabolic substrates such as ^11^C-acetate and palmitate analogue 14 (R,S)-[^18^F]fluoro-6-thiaheptadecanoic acid (^18^F-FTHA) [29].

Using the stromal vascular fraction from the raccoon dog subcutaneous adipose tissue and a protocol for culturing and differentiation of adipocytes from preadipocytes, our in vitro results demonstrated that *UCP1* expression was not induced in response to sympathetic stimuli from NA or a selective β3-AR agonist [30]. However, we did observe significant increases in raccoon dog adipocyte *UCP1* levels in response to a nonselective β-AR agonist, isoprenaline, which has a higher affinity to the β1- and β2-receptors, than to β3-receptors. The *UCP1* increase with the nonselective β-AR agonist and the lack of response to a selective β-AR agonist suggest, that similarly to the guinea pig [31], the raccoon dog might lack functional β3-ARs, and the observed increase in *UCP1* expression could be the result of β1- and β2-ARs stimulation. The *UCP1* increase is a likely by-product of the activated lipolytic and β-oxidation pathways to utilize WAT as an energy source [32] and a remnant that may not have a physiological relevance for heat production in the raccoon dog.

In addition to β-AR agonists, we tested the effects of irisin, natriuretic peptides (ANP, BNP), PPARα agonist (fenofibrate), apelin, and lactate on *UCP1* induction and beige adipocyte transformation in raccoon dog adipocytes. Irisin is an adipomyokine [33], cleaved from its parent molecule FNDC5, a G-protein coupled transmembrane protein found in adipose and muscle tissue [34]. Cold environmental temperature and exercise is thought to cleave irisin from FNDC5 [35], which then is transported via circulation to adipose tissue where it induces the recruitment of the beige adipocytes and their thermogenic function [33]. Another adipose tissue-derived factor that has been found to induce WAT browning, is apelin [36,37]. Apelin participates in energy metabolism and promotes insulin sensitivity and glucose utilization [38,39]. Than et al. discovered that apelin treatment increased *UCP1* expression in human white adipocytes and in 3T3-L1 cells with increased mitochondrial respiration, and induced beige adipocyte morphology [36]. In addition, various PPAR agonists induce *UCP1* expression and beige adipose tissue recruitment in mouse and human WAT [40,41]. Fenofibrate, a PPARα agonist, induces beige adipocyte formation in mouse WAT. Cardiac natriuretic peptides have WAT browning effects via various mechanisms. ANP treatment dose-dependently increases *UCP1* and PPARγ coactivator-1α (PGC-1α) expression in human and mouse adipocytes [42,43]. In addition to peptides, certain metabolite products are linked to browning of WAT. Lactate increases *UCP1* expression in WAT by modifying intracellular redox reactions [44]. In our raccoon dog adipocytes cultures, these various peptides and substances did not increase *UCP1* expression and beige adipocyte morphology, while the nonselective β-AR agonist isoprenaline did. In contrast, we observed that lactate significantly stimulated *UCP1* expression in the mouse 3T3-L1 adipocytes. These findings indicate that adipocytes in mice and other small rodents are more susceptible to WAT browning as an additional form to produce heat, due to the increased heat dissipation and energy expenditure from their body surface and smaller body to surface volume ratio [45,46].

In addition to the UCP1-dependent thermogenesis via BAT and beige adipose tissue, recent discoveries indicate possible alternative UCP1-independent pathways for thermogenesis [47]. Skeletal muscle is thought to participate in UCP1-independent thermogenesis through the function of the contractile activity in shivering thermogenesis and the function of SERCA [48]. Also, brown and beige adipose tissues have been found to express UCP1-independent thermogenesis [47]. One of the suggested mechanisms for UCP1-independent heat production is related to a cycling mechanism for ATP-dependent Ca^2+^ cycling via SERCA2b selectively in the beige adipose tissue [49]. Creatinine-substrate cycling stimulates mitochondrial respiration and contributes to the thermogenic pathway [50]. As the raccoon dog appears to lack BAT and beige adipose tissue UCP1 response, it is possible that the raccoon dog might utilize some of these UCP1-independent mechanisms to contribute to the heat production in the cold.

Our results suggest that adult raccoon dogs do not have active BAT, and WAT is not recruited into UCP1-dependent thermogenesis via beige cells. Its’ fur coat, insulating WAT, and muscle shivering [51,52], are likely to provide adequate heat for maintaining body temperature in the raccoon dog.

## 4. Materials and Methods

### 4.1. Animals and Sampling

The experiment was approved by the National Committee for Animal Experimentation (license no. ESLH-2008-09952/Ym-23). PET/CT imaging was conducted to three wild-born raccoon dogs (*n* = 3, male, adult, weight 10 ± 1.5 kg) caught from the Evo region in Southern Finland and imaged during winter (February). Additional fresh adipose tissue samples were collected from six farm-bred raccoon dogs (*n* = 6, male, adult, weight 10.1 ± 0.9 kg) reared at a farm in Kalajoki, Finland in the winter (March) for the in vitro studies.

### 4.2. Imaging Study: ^18^F-FDG PET/CT

For PET/CT imaging, three raccoon dogs were anesthetized using a combination of dexmedetomidine (25 µg/kg), midazolam (0.45 mg/kg), and butorphanol (0.25 mg/kg). The raccoon dogs were intravenously injected with 89 ± 6.6 MBq ^18^F-FDG and whole-body PET/CT data were acquired using a clinical GE Discovery VCT system (General Electric Medical Systems, Milwaukee, WI, USA) for the two first animals for 120 min and for the third animal along with one of the first two for 60 min starting from the time of the injection. Serial venous blood samples were collected after injection of ^18^F-FDG. Plasma glucose was determined (Analox GM7 Analyzer; Analox Instruments, London, UK). CT was performed for attenuation correction and for the anatomical localization of ^18^F-FDG uptakes. The PET data were reconstructed using a 3D maximum likelihood ordered subsets expectation maximization algorithm (VUE Point, General Electric Medical Systems, Chicago, IL, USA). Quantitative PET analysis was performed using Carimas 2.10 software (Turku PET Centre, Turku, Finland). The regions of interests (ROIs) were defined in heart, liver, lymph nodes (forelimb, hindlimb and neck), muscle, white adipose tissue, kidney, and urinary bladder using CT as the anatomical reference. The radioactivity concentrations were corrected for injected radioactivity dose and decay, and the results were presented as SUV. Time-activity curves (TACs) extracted from dynamic PET images were used for presenting kinetics of ^18^F-FDG uptake. The kinetic influx constant (K_i_) of ^18^F-FDG was determined using the Patlak analysis of TACs generated for each ROI. The graphic analysis approach, as described by Patlak et al. [28], was used to analyze the uptake kinetics of ^18^F-FDG. The rate of glucose uptake was calculated by multiplying the K_i_ of ^18^F-FDG by the mean plasma glucose concentration during ^18^F-FDG imaging, to obtain an index kinetic influx constant, K_i_[G]. The raccoon dogs were sacrificed after the PET/CT imaging under deep anaesthesia in accordance with the American Veterinary Medical Association’s Guidelines on Euthanasia with an overdose of pentobarbital sodium (Euthasol Vet 400 mg/mL, Le Vet. Pharma, Netherlands). Various tissue samples were excised and weighted, and total radioactivity was measured using a gamma counter (1480 Wizard 3”, Perkin Elmer, Turku, Finland). The ex vivo results were expressed as SUV. After the PET/CT imaging, tissue samples were formalin-fixed, paraffin-embedded, and cut into 20 µm sections for hematoxylin-eosin staining. Tissue sections were studied for morphology under a light microscope.

### 4.3. In Vitro Studies

Adipose tissue samples for the in vitro culturing of raccoon dog adipocytes were obtained from six farm-raised raccoon dogs. The farmed raccoon dogs were housed in roofed enclosures under natural temperature and photoperiod and had ad libitum access to commercial fur animal diet and water. The animals were euthanized with an electric shock according to the legislation of the Council of the European Union (1993) and the Finnish Act on Animal Experimentation (62/2006). The fresh adipose tissue samples collected from subcutaneous adipose tissue were immediately immersed in 1% penicillin-streptomycin (Thermo Fisher Scientific, Waltham, MA, USA) PBS solution and transferred on ice to cell culture laboratory for the extraction of the stromal vascular fraction and further cell culture treatments. Mouse 3T3-L1 cell line (ATCC, Manassas, VA, USA) was used as positive control; cells were differentiated to adipocytes and used as methodological control for the cell culture treatments.

#### 4.3.1. Differentiating Primary Adipocytes and Browning Inducing Peptide Treatments

The raccoon dog subcutaneous adipose tissue was dissected and minced into smaller pieces [53]. The tissue fragments were washed with PBS solution containing 1% penicillin-streptomycin. The tissue was digested with collagenase type I (1.5 mg/mL, Sigma Aldrich, St. Louis, MO, USA) in PBS containing 1% bovine serum albumin (BSA) for 60 min at 37°C with intermittent shaking. Digested fat tissues were filtered through a nylon mesh (250 micron) and centrifuged at 300× *g* for 10 min. Supernatant containing mature adipocytes were discarded and the pelleted stromal vascular cell fractions (SVF) were washed twice with PBS containing 2.5% BSA. The cells were suspended in DMEM containing 10% fetal bovine serum (FBS), 2 mmol/L glutamine, and 50 U/mL penicillin and streptomycin (Thermo Fisher Scientific, Waltham, MA, USA), and cultured in CO_2_ incubator at 37 °C. The cells were grown up to near confluency and differentiated into adipocytes using adipogenic induction medium (AIM) for 3 days. After 3 days, AIM was switched to adipogenic maintenance medium (AMM) for 3 days. This cycle was then repeated until day 13.

For the testing of the different browning factors, individual cell wells were co-incubated with one of the factors from day 7 onwards, the AIM (day 7) and AMM (day 10): selective β3-adrenergic receptor agonist 1 µmol/L (ZD 7114 hydrochloride, Bio-Techne Brands Tocris, Minneapolis, MN, USA) and noradrenaline 1 µmol/L (Sigma Aldrich, Burlington, MA, USA), isoprenaline 1 µmol/L (Sigma Aldrich, Burlington, MA, US), ANP 100 nmol/L, BNP 100 nmol/L (from department peptide library), lactate 1 µmol/L (Sigma Aldrich, Burlington, MA, US) [44], apelin-12 100 nmol/L (from department peptide library) [36,54], irisin 163 µmol/L (synthetized recombinant irisin, described later) [33,55], fenofibrate 100 µmol/L (Sigma Aldrich, Burlington, MA, US), and individually tested for their chronic effect on beige induction. A recombinant irisin was produced in *Escherichia coli*, purified, and the product confirmed with mass spectrometry. Apelin-12 was synthesized by solid-phase synthetic technique using an automatic peptide synthesizer (Model 431, Applied Biosystems/Thermo Fisher Scientific, Waltham, MA, USA) and purified by high-performance liquid chromatography (HPLC) after deprotection [54].

Acute study: Cells were incubated with different dosages (0.1 µmol/L, 1 µmol/L, 10 µmol/L, 100 µmol/L, and 1000 µmol/L isoprenaline) up to 24 h. 

Chronic study: Cells were incubated with 1 µmol/L isoprenaline up to 7 days. Furthermore, in additional wells, 100 µmol/L isoprenaline was added on day 12, 24 h prior to mRNA extraction as positive control [24]. PBS was used as a control in all treatments. 

On day 13, adipogenic potentials were analyzed by the Oil red O staining (Sigma Aldrich, Burlington, MA, US) and total RNA was isolated for *UCP1* expression analysis as adipogenic beige marker. In addition to the raccoon dog samples, mouse 3T3-L1 was methodological control for the cell culture treatments as described in the Supplementary Materials.

#### 4.3.2. Adipocyte *UCP1* Expression

The expression of BAT and beige adipose tissue-specific thermogenic *UCP1* was determined by quantitative real-time PCR from the treated raccoon dog adipocytes and mouse 3T3-L1 cells. Total RNA was extracted from the cell culture samples with NucleoSpin^®^ RNA Plus (Macherey-Nagel, GmbH & Co. KG, Düren, Germany) and RNeasy Mini Kit (Qiagen, Hilden, Germany) assays according to the manufacturer’s instructions. SensiFAST™ cDNA Synthesis Kit (Bioline, Meridian Biosciences, Cincinnati, OH, USA) was utilized in synthesizing cDNA from 500 ng of RNA from raccoon dog adipocytes and from 1 µg of RNA from mouse 3T3-L1 adipocytes. The TAQMAN Eagletaq (Roche Holding AG, Basel, Switzerland) real-time PCR reactions were performed by ABI-PRISM 7300 sequence detection system (Thermo Fisher Scientific, Waltham, MA, USA) in a total volume of 50 µL. The samples were analyzed as duplicates along with the internal control gene beta-2 microglobulin (*B2M*) as housekeeping gene [56] in a 96-well plate. Sterile water was used as a negative control. The results were analyzed and calculated according to the manufacturer instructions. TAQMAN oligos (Thermo Fisher Scientific, Waltham, MA, USA) optimized and tested for the sequenced domestic dog (*Canis lupus familiaris*) genome were used for the analysis of raccoon dog adipocyte *UCP1* (Cf02622091) and *B2M* (Cf02659079). Specific mouse TAQMAN oligos (Thermo Fisher Scientific, Waltham, MA, USA) were also used for detecting mouse UCPI (Mm01244861) and B2M (Mm00437762) expressions in the 3T3-L1 adipocytes.

### 4.4. Statistical Analyses

The multiple comparisons were performed with the one-way analysis of variance (ANOVA) followed by Tukey’s post hoc test using the IBM SPSS Statistics 21 Data Editor software (IBM, Armonk, NY, USA) for the gene expression analyses. In the results, a *p*-value less than 0.05 was considered statistically significant and the results were presented as mean ± SD.

## 5. Conclusions

The ^18^F-FDG PET/CT imaging and histopathological analysis of the adult raccoon dog during winter indicate the absence of classical BAT. In vitro cell culturing of raccoon dog adipocytes and treatment with a set of browning agents, including selective β3-AR agonist, did not induce the recruitment of beige adipocytes according to thermogenic *UCP1* expression and cell morphology. The raccoon dog exhibits resilience to cold weather without resorting to thermogenesis via BAT or WAT browning.

## Figures and Tables

**Table 1:** Representative plasma glucose-corrected net influx rate (Ki) of ^18^F-FDG uptake obtained by dynamic PET and Patlak analysis (please see the method section for detailes) from one of the raccoon dogs.

Organ	K_i_ (mL/mL·min)
Brain	0.155
Heart	0.251
Liver	0.013
Lymph node forelimb	0.080
Lymph node hindlimb	0.087
Lymph node neck	0.105
Muscle	0.022
White adipose tissue	0.011

## Data Availability

Not applicable.

## Supplementary Materials

The following are available online at https://www.mdpi.com/article/10.3390/ijms22179623/s1, Figure S1: Histological hematoxylin-eosin staining, Figure S2: Relative expression of UCP1 in mouse 3T3-L1 cells.
