# Supplementary material for: Role of Brown and Beige Adipose Tissues in Seasonal Adaptation in the Raccoon Dog (Nyctereutes procyonoides)
Source: Int J Mol Sci. 2021 Sep 6;22(17):9623. doi: 10.3390/ijms22179623 (PMC8431801; doi:10.3390/ijms22179623)
Supplement: Supplementary file 1 [file ijms-22-09623-s001.zip › ijms-1277037-supplementary.pdf]

Supplement

Browning capacity of mouse white preadipocyte cell line 3T3-L1 as positive control

Mouse 3T3-L1 cells were differentiated to adipocytes and beige adipocyte differentiation potential was tested using different beige adipose tissue inducers similarly to that of raccoon dog primary adipocytes as described (4.3.1. Differentiating primary adipocytes and browning inducing peptide treatments). Briefly, 1 × 105 cells were plated on to 6-well plates, differentiation was initiated by switch to induction medium for 2 days followed by the 3 days maintenance medium. The tested browning inducing peptides and chemicals were supplemented in maintenance medium for the treatment. Irisin/FNDC5 (Abcam, Cambridge, UK) 50 nmol/L [1,2] and apelin-12 100 nmol/L [3], were tested for 4 days. Lactate 50 mmol/L [4] and bone morphogenic protein 7 (BMP7, R&D Systems, Minneapolis, Minnesota, USA) 3.3 nmol/L [5], were tested for 2 days. Isoprenaline 5 µmol/L was tested for 15-hour treatment [6]. Adipocyte differentiation was confirmed with Oil red O staining and RNA was isolated from the cells.

Supplementary figures

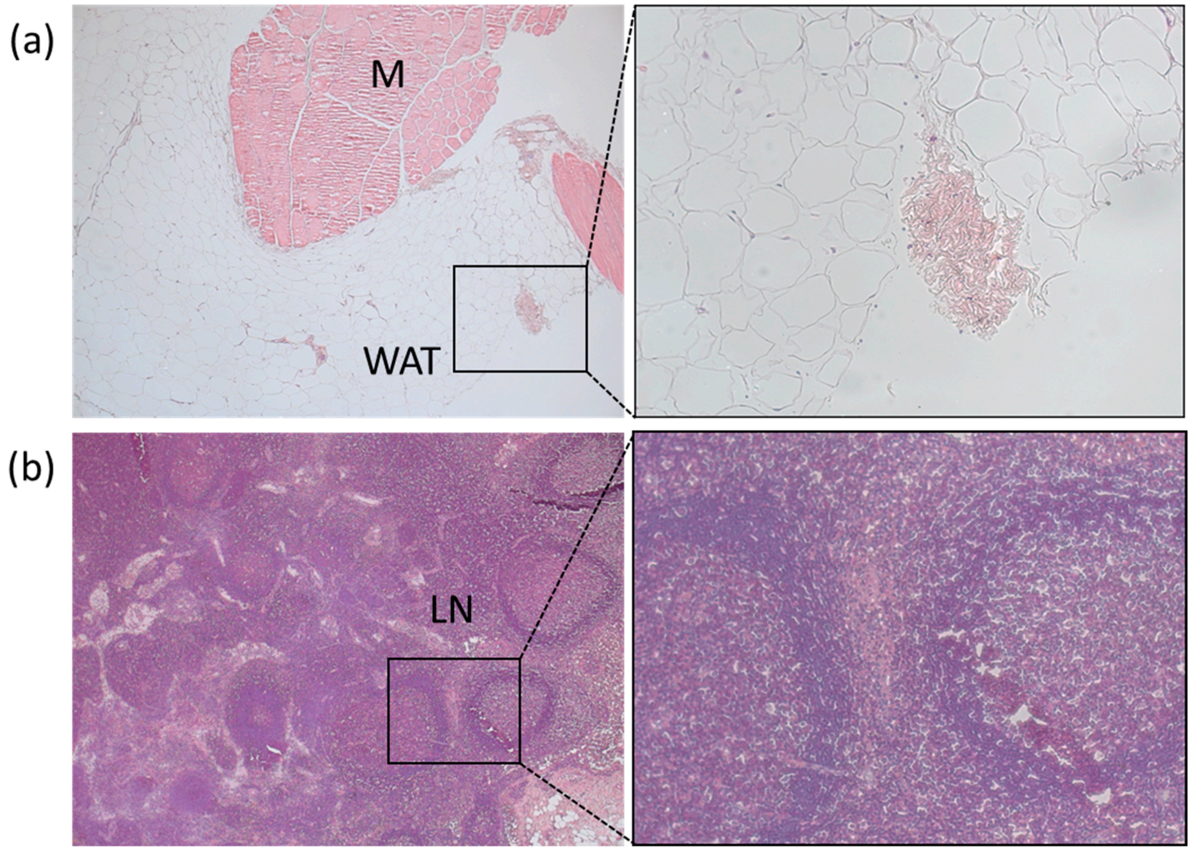

Figure S1: Histological hematoxylin-eosin staining. Histological hematoxylin-eosin staining of the active areas detected for FDG retention of one of the wild-born raccoon dogs imaged with PET/CT. The sample turn out to be muscle and white adipose tissue **a**), and the other sample was lymph node **b**). Paraffin-embedded tissue sections at × 5 original magnification. The images on the left show high-power views (× 20 magnification) of the area within the black rectangle on the right images. M = muscle, WAT = white adipose tissue, LN =lymph node.

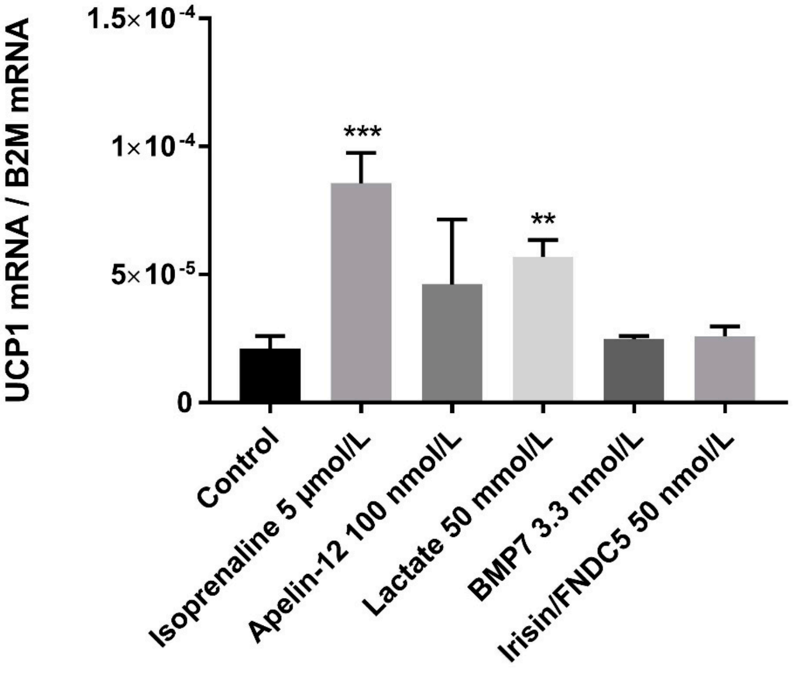

Figure S2. Relative expression of UCP1 in mouse 3T3-L1 cells. The cells ($n = 4$) were treated with 5 µmol/L isoprenaline for 15 hours as positive control; 100 nmol/ apelin-12 and 50 mmol/L lactate for 4 days and 3.3 nmol/L BMP7 and 50 nmol/L irisin/FNDC5 for 2 days. Control cells were treated with PBS. UCP1 expressions are normalized to the expression of B2M and the result values are presented as mean ± SD. Statistically significant differences are indicated by ** $p \leq 0.01$ and *** $p \leq 0.001$.

Supplementary table

Table S1: AIM and AMM reagents.

**Adipogenic Media**	**Reagents**
AIM (Adipogenic induction media)	-DMEM (Sigma Aldrich, St. Louis, Missouri, USA) with 4500 mg/mL glucose<br>-10% FBS, 1% penicillin/streptomycin (Thermo Fisher Scientific, Waltham, Massachusetts, USA)<br>-1 µmol/L dexamethasone (Sigma Aldrich)<br>-0.5 mmol/L methylisobutylxanthine (Sigma Aldrich)<br>-1.7 µmol/L insulin (Novo Nordisk, Bagsvaerd, Denmark)<br>-200 µmol/L indomethacin (Sigma Aldrich)<br>-1 µmol/L rosiglitazone (Sigma Aldrich)<br>-1 nmol/L 3.3′,5-L-thyronine sodium salt (Sigma Aldrich)
AMM (Adipogenic maintenance media)	-DMEM (Sigma Aldrich) with 4500 mg/mL glucose<br>-10% FBS, 1% penicillin/streptomycin (Thermo Fisher Scientific)<br>-1.7 µmol/L insulin (Novo Nordisk)

	-1 μmol/L rosiglitazone (Sigma Aldrich)<br>-1 nmol/L 3.3',5-L-thyronine sodium salt (Sigma Aldrich)

References

1. Boström, P.; Wu, J.; Jedrychowski, M.P.; Korde, A.; Ye, L.; Lo, J.C.; Rasbach, K.A.; Boström, E.A.; Choi, J.H.; Long, J.Z.; Kajimura, S.; Zingaretti, M.C.; Vind, B.F.; Tu, H.; Cinti, S.; Højlund, K.; Gygi, S.P.; Spiegelman, B.M. A PGC1-a-dependent myokine that drives brown-fat-like development of white fat and thermogenesis. *Nature* **2012**, *481*, 463-468.

2. Li, H.; Zhang, Y.; Wang, F.; Donelan, W.; Zona, M.C.; Li, S.; Reeves, W.; Ding, Y.; Tang, D.; Yang, L. Effects of irisin on the differentiation and browning of human visceral white adipocytes. *Am J Transl Res* **2019**, *11*, 7410-7421.

3. Than, A.; He, H.L.; Chua, S.H.; Xu, D.; Sun, L.; Leow, M.K.; Chen, P. Apelin Enhances Brown Adipogenesis and Browning of White Adipocytes. *J Biol Chem* **2015**, *290*, 14679-14691, DOI 10.1074/jbc.M115.643817 [doi].

4. Carriere, A.; Jeanson, Y.; Berger-Muller, S.; Andre, M.; Chenouard, V.; Arnaud, E.; Barreau, C.; Walther, R.; Galinier, A.; Wdziekonski, B.; Villageois, P.; Louche, K.; Collas, P.; Moro, C.; Dani, C.; Villarroya, F.; Casteilla, L. Browning of white adipose cells by intermediate metabolites: an adaptive mechanism to alleviate redox pressure. *Diabetes* **2014**, *63*, 3253-3265, DOI 10.2337/db13-1885 [doi].

5. Tseng, Y.H.; Kokkotou, E.; Schulz, T.J.; Huang, T.L.; Winnay, J.N.; Taniguchi, C.M.; Tran, T.T.; Suzuki, R.; Espinoza, D.O.; Yamamoto, Y.; Ahrens, M.J.; Dudley, A.T.; Norris, A.W.; Kulkarni, R.N.; Kahn, C.R. New role of bone morphogenetic protein 7 in brown adipogenesis and energy expenditure. *Nature* **2008**, *454*, 1000-1004, DOI 10.1038/nature07221 [doi].

6. Miller, C.N.; Yang, J.Y.; England, E.; Yin, A.; Baile, C.A.; Rayalam, S. Isoproterenol Increases Uncoupling, Glycolysis, and Markers of Beiging in Mature 3T3-L1 Adipocytes. *PLoS One* **2015**, *10*, e0138344, DOI 10.1371/journal.pone.0138344 [doi].
